# Experiment and Simulation Study on the Crashworthiness of Markforged 3D-Printed Carbon/Kevlar Hybrid Continuous Fiber Composite Honeycomb Structures

**DOI:** 10.3390/ma18010192

**Published:** 2025-01-05

**Authors:** Jinlong Ju, Nana Yang, Lei Yu, Zhe Zhang, Hongyong Jiang, Wenhua Wu, Guolu Ma

**Affiliations:** 1 College of Shipbuilding Engineering, Harbin Engineering University, Harbin 150001, China; jujinlong@hrbeu.edu.cn (J.J.); yangnana@hrbeu.edu.cn (N.Y.); zhezhezhe1@hrbeu.edu.cn (Z.Z.); 2 China Aerodynamics Research and Development Center, Mianyang 621000, China; yulei200508@163.com; 3 School of Mechanical Engineering and Electronic Information, China University of Geosciences, Wuhan 430074, China; 4 Key Laboratory of Testing Technology for Manufacturing Process of Ministry of Education, Southwest University of Science and Technology, Mianyang 621010, China; maguolu578@163.com

**Keywords:** hybrid fiber composite, honeycomb, crashworthiness, 3D printing, Markforged

## Abstract

Fiber hybridization can effectively solve the localized brittle fracture problem of composite honeycomb, but the interaction between different fibers leads to a very complex failure mechanism. Hence, 3D-printed hybrid continuous fiber composite honeycombs with a combination of carbon and Kevlar fibers are designed to study the structural failure behaviors by the experiment and simulation method. The experimental samples, including Onyx, carbon, Kevlar, carbon/Kevlar, and Kevlar/carbon composites, are fabricated based on Markforged 3D printing technology, and the crushing tests are conducted to evaluate the failure behaviors. An equivalence finite element modeling method to replace the heterogeneous microstructure of hybrid composites is proposed to analyze the failure behaviors. Results indicate that carbon/Kevlar honeycomb exhibits the highest energy absorption and cost effectiveness, while CFRP honeycomb demonstrates the highest load-carrying capacity. It is found that carbon/Kevlar and Kevlar/carbon honeycombs have significant hybrid effects compared to single-fiber honeycombs, which also reveals the hybrid mechanisms between carbon and Kevlar fibers. Furthermore, the Onyx honeycomb, lacking long fibers, exhibits brittle collapse, whereas other honeycombs show ductile collapse due to the presence of Kevlar fibers. Combining the simulation studies, the damage evolution mechanisms of honeycombs, including fiber/matrix tension and compression, shear damage, interface damage, etc., are further revealed. This work provides valuable insights into the design and failure analysis of 3D-printed hybrid fiber composite honeycombs.

## 1. Introduction

The advancement in additive manufacturing technologies has revolutionized the design and fabrication of complex structures, enabling the production of components with intricate geometries that were previously unattainable through traditional manufacturing methods [1]. Fused Deposition Modeling (FDM) is a widely used additive manufacturing technique [2]. In FDM 3D printing, a thermoplastic filament is fed through a heated nozzle. The nozzle heats the filament to its melting point and then deposits it layer by layer according to the pre-designed model [3]. This layer-by-layer deposition process allows for the creation of complex three-dimensional geometries. For continuous fiber composites in FDM, it offers many unique advantages. The continuous fibers can be incorporated into the thermoplastic matrix during the extrusion process [4]. This enables the reinforcement of the printed structure, enhancing its mechanical properties, such as strength and stiffness. The ability to precisely control the fiber orientation and distribution within each layer provides an opportunity to optimize the performance of the final structure. For example, by aligning the fibers along the direction of the main stress, the load-bearing capacity of the structure can be significantly improved. Additionally, FDM technology allows for relatively easy customization and rapid prototyping, which is beneficial for fabricating complex structures with different cell sizes and geometries to meet specific application requirements [5].

Recently, the utilization of 3D-printed structures for energy absorption purposes has garnered considerable attention due to their exceptional strength-to-weight ratio rendering them advantageous for applications necessitating lightweight structures, notably within aerospace industries [6,7]. Incorporating continuous fibers into these structures by 3D printing further augments their mechanical prowess, particularly enhancing energy absorption and impact resistance [8]. Notably, Dou et al. [9] investigated the in-plane compression characteristics of 3D-printed continuous carbon-fiber-reinforced composite honeycombs, revealing substantial enhancements in properties in contrast to PLA honeycombs. Likewise, Zeng et al. [10] conducted an empirical investigation on the compression behavior of 3D-printed continuous-fiber-reinforced composite honeycombs, elucidating collapse modes and energy absorption mechanisms. Pirouzfar et al. [11] employed 3D printing to fabricate honeycomb cores and explored the influence of geometrical parameters on the flexural properties of honeycomb sandwiches. Additionally, Tian et al. [12] and Zhao et al. [13] showcased notable enhancements in the mechanical properties of fiber-reinforced composite auxetic honeycomb structures, albeit with relatively complex failure mechanisms. Lu et al. [14] demonstrated that the incorporation of fibers led to an 86.3% increase in compressive stiffness and a 100% increase in energy absorption in auxetic composites with a mere 6% increase in mass. Furthermore, Qin et al. [15] observed a twofold enhancement in modulus and energy absorption through fiber reinforcement of chiral composite structures.

The aforementioned studies have extensively examined the class of 3D-printed composite energy-absorbing structures, consistently demonstrating that fiber-reinforced composites exhibit substantial improvements in terms of crashworthiness. These enhancements are primarily attributed to the ability of fibers to effectively impede matrix crack propagation, thereby enhancing the fracture toughness of the matrix. Nonetheless, the majority of research has concentrated on 3D-printed carbon fiber composite energy-absorbing structures. These structures are susceptible to brittle fracture during the damage process, leading to abrupt declines in load-bearing capacity, which is detrimental to sustained energy absorption [16,17].

Carbon fibers are known for their high stiffness and strength, while Kevlar fibers are renowned for their superior toughness and energy dissipation properties [18,19]. By integrating these materials into a hybrid composite, it is possible to harness the advantages of both fiber types, potentially yielding structures with enhanced crashworthiness. Therefore, it is crucial to incorporate high-toughness fibers alongside high-strength fibers to ameliorate the failure behavior of carbon fiber composite energy-absorbing structures, thereby enhancing their energy absorption capabilities. Here are some studies on 3D printing process parameters and the structural mechanics of hybrid fiber composites. Ding et al. [20] investigated the effects of process parameters, such as layer thickness and path width, in the 3D printing of pre-impregnated continuous carbon, glass fibers, and their hybrid composites. Their research demonstrated that the optimization of these parameters could lead to a significant improvement in the overall performances of printed parts. Some studies also reported research on the FDM 3D printing of hybrid fiber composites, which indicates that the hybrid fiber 3D printing process is more complex and still faced issues such as differences in the impregnation of different fibers in PLA at the same temperature [21,22]. Zia et al. [23] investigated the mechanical and energy absorption behaviors of 3D-printed continuous carbon/Kevlar-hybrid-thread-reinforced PLA composites, demonstrating that hybridization can achieve superior comprehensive properties. Morales et al. [24] examined the quasi-static and dynamic crush behavior of 3D-printed thin-walled profiles reinforced with continuous carbon and glass fibers with a Markforged Two printer. Wang et al. [25] studied the mechanical properties of polyamide-based composites with hybrid continuous fibers produced via 3D printing, revealing concurrent improvements in both rigidity and energy absorption. Collectively, these studies underscore that mixing high-strength and high-toughness fibers can produce synergistic effects, significantly enhancing the mechanical properties of the resulting composites.

Despite the potential benefits, the mechanical mechanisms (including energy absorption, failure modes, and hybrid interactions) of 3D-printed hybrid composite energy-absorbing structures remain insufficiently understood. Conventional experimental methods for evaluating crashworthiness are frequently both time-intensive and expensive [26]. Consequently, an integrated experimental and simulation approach is imperative to achieving a thorough understanding of the energy absorption characteristics of these advanced materials and structures. Finite element simulations, in particular, are instrumental in predicting the behavior of complex structures under crushing loads, facilitating the optimization of design parameters prior to physical testing [27,28]. This dual approach not only enhances efficiency but also provides a more nuanced understanding of the underlying mechanical mechanisms, thereby advancing the development of superior energy-absorbing structures.

This study aims to investigate the crashworthiness of 3D-printed carbon/Kevlar hybrid continuous fiber composite honeycomb structures through both experimental testing and finite element simulations. Several honeycomb structures are proposed and their crushing behaviors, energy absorption capabilities and cost-effectiveness are evaluated. Then, the hybrid mechanisms of hybrid fiber composite honeycomb are revealed to explain the improvements in crashworthiness. Through the experimental characterization and simulation, the crushing failure process and failure modes are analyzed to reveal the structural failure mechanisms. Finally, the additional structural parameters in carbon/Kevlar (CF/KFRP) honeycomb are predicted and analyzed.

## 2. Designs, Materials and Methods

### 2.1. Hybrid Honeycomb Design

**Figure 1:**
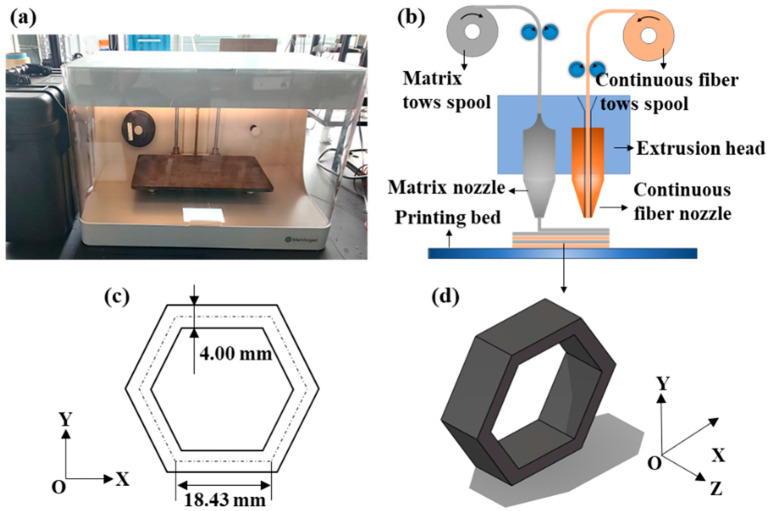
(**a**) Markforged Two 3D printer. (**b**) Markforged 3D printing principles. (**c**) Dimensions of honeycomb. (**d**) Three-dimensional diagram of honeycomb.

Figure 1 illustrates the Markforged Two 3D printer (Mark-forged® Inc., Watertown, MA, USA), its 3D printing principles, and the dimensions of the honeycomb structure. The study encompasses both single-fiber and hybrid fiber composite honeycomb structures, including configurations of Carbon-Fiber-Reinforced Polymer (CFRP), Kevlar-Fiber-Reinforced Polymer (KFRP), Carbon-Fiber/Kevlar-Fiber-Reinforced Polymer (CF/KFRP), and Kevlar-Fiber/Carbon-Fiber-Reinforced Polymer (KF/CFRP). Carbon fibers are known for their exceptional tension strength and stiffness, while Kevlar fibers provide excellent toughness and energy-absorption capabilities [29,30]. The combination of these fibers in hybrid composites can potentially exploit the advantageous properties of both materials, leading to superior performance characteristics.

**Figure 2:**
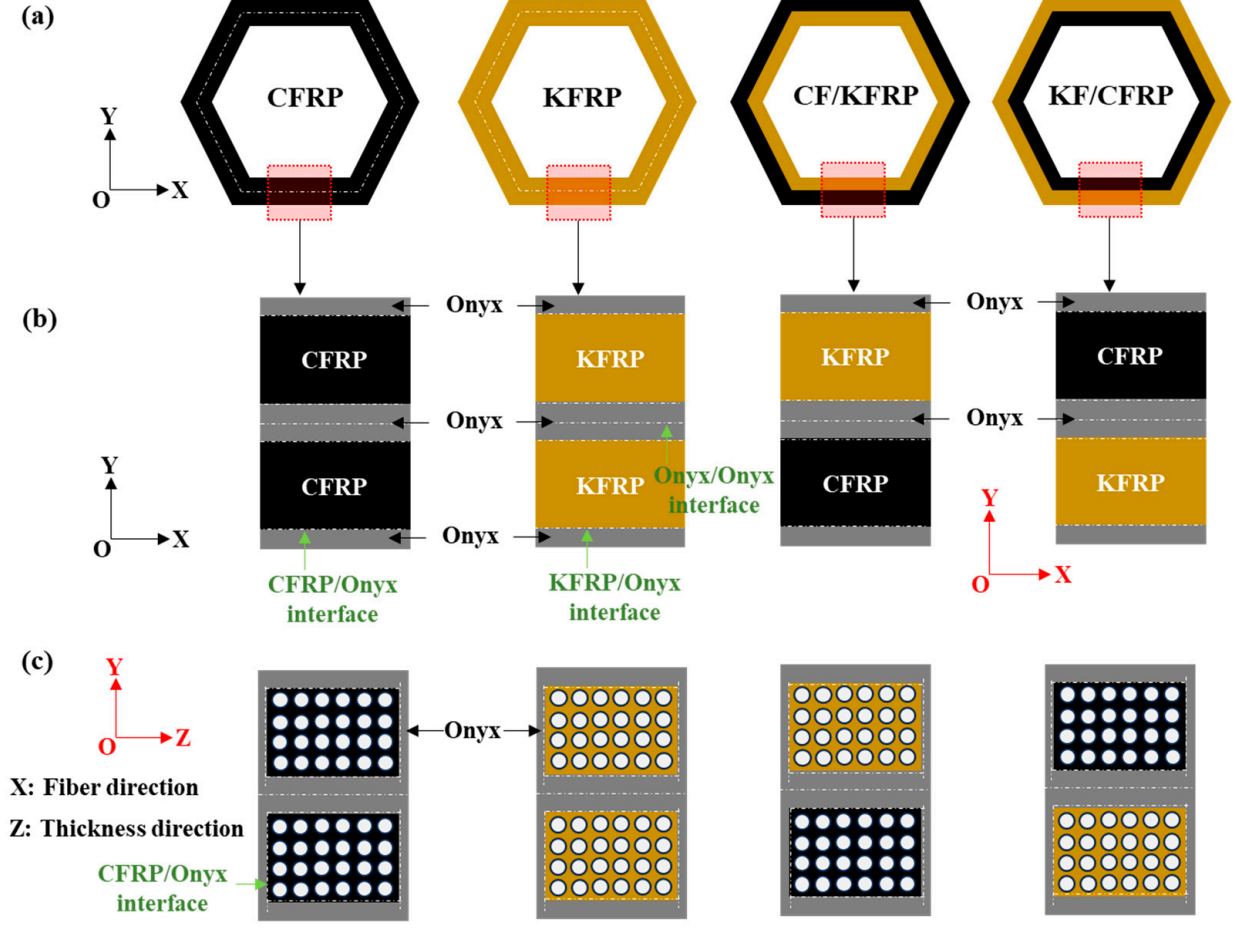
Design of composite honeycomb structures and the partial schematic diagram. (**a**) Stacking sequence design at the macro-scale. (**b**) Details of layup at XOY- CSYS. (**c**) Details of layup at YOZ- CSYS.

Figure 2 shows the design of composite honeycomb structures and the partial schematic diagram. As shown in Figure 2a, the single-fiber composite honeycomb structures, specifically CFRP and KFRP, serve as the reference for comparative analysis. For the hybrid structures, in the CF/KFRP configuration, the carbon fiber layer is positioned on the exterior, while, in the KF/CFRP configuration, the Kevlar fiber layer occupies the outermost position. This layup is informed by previous studies which have shown that placing the stiffer carbon fiber layers on the outside can enhance the structural stiffness and that Kevlar layers on the exterior can improve impact resistance [31]. Due to the constraints imposed by the Markforged 3D printer (Figure 1a), several Onyx layers are incorporated to safeguard the fiber layers, as depicted in Figure 2b. Onyx, a composite material consisting of Nylon mixed with chopped carbon fibers, is known for its good mechanical properties and printability [32]. Consequently, interfaces between CFRP/Onyx, KFRP/Onyx, and Onyx/Onyx are formed and can be observed in the XOY coordinate system (CSYS), and the properties and role of Onyx will be further elaborated in the subsequent section. The presence of Onyx layers provides a protective barrier that mitigates potential damage to the fiber layers during the printing process [33].

Moreover, in the YOZ coordinate system (CSYS), it is evident that the fiber layers are enveloped by Onyx on both sides along the thickness direction, as shown in Figure 2c. This encapsulation of fiber layers within Onyx can lead to improved load distribution and damage tolerance, as the Onyx layers help to absorb and dissipate energy under mechanical loading [34]. These detailed configurations and their implications on the mechanical behavior of composite honeycomb structures will be comprehensively analyzed and discussed in the following sections, emphasizing the significance of the synergistic effects of combining different fiber types.

### 2.2. Materials and Additive Manufacturing

**Figure 3:**
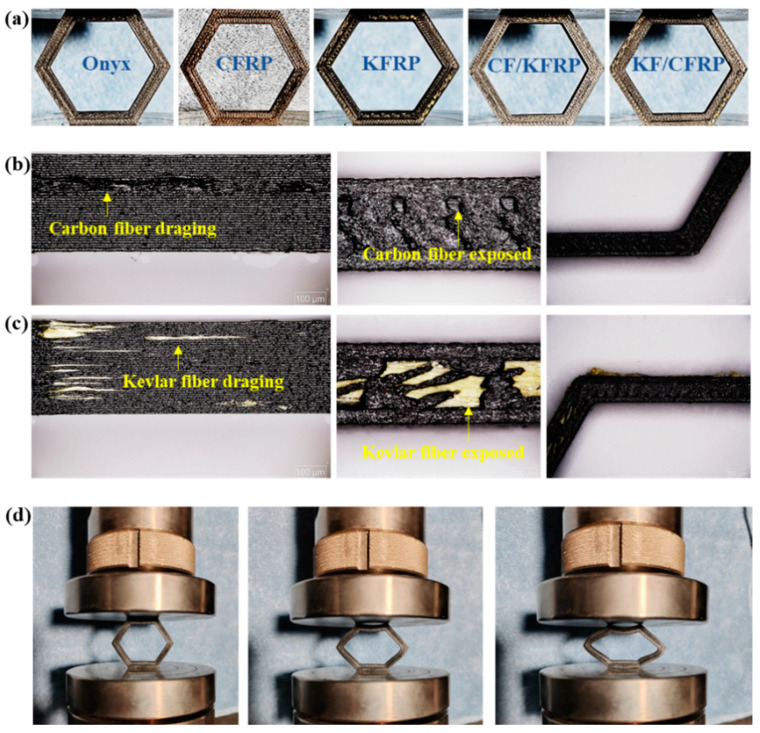
(**a**) The 3D-printed experimental samples. (**b**,**c**) Printing defects characterized by a super-depth-of-field microscope. (**d**) Quasi-static crushing test.

In this study, Fused Filament Fabrication (FFF)-based 3D printing technology was utilized to fabricate the experimental specimens, as illustrated in Figure 3a. The 3D printer used was the Markforged Two (Markforged^®^ Inc., Watertown, MA, USA), and the filaments were also sourced from Markforged. The primary parameters of three filaments are detailed in Table 1, including continuous carbon-fibers/Kevlar-fibers embedded in a PA6 matrix (cCF/O or cKF/O) and Polyamide-6 (PA6) reinforced with short carbon fibers (Onyx). Following the printing process, different fiber layers were thermally cured with high-strength epoxy resin to produce the final 3D-printed continuous carbon/Kevlar hybrid-fiber-reinforced composite honeycomb structures. The specific details of the specimens are as follows: 4.00 mm in wall thickness, 18.43 mm in edge length (along the center line), and 4.80 mm in width, as shown in Figure 1c. To ensure a consistent proportion of fibers in each specimen, different layers of neat Onyx were strategically placed during the printing of continuous carbon fiber layers (cCF/O) and continuous Kevlar fiber layers (cKF/O). Specifically, two layers of neat Onyx were placed atop the cCF/O and cKF/O layers in the CF/KFRP or KF/CFRP configuration. Then, the slicing software (Eiger 3.20.13, from Markforged^®^ Inc.) was adopted to finish the slicing of the honeycombs. After the 3D printing process was completed, the CFRP and KFRP needed to be bonded together with high-strength adhesive. ARALDITE^®^ AW 106 CI epoxy resin (Huntsman Petrochemical LLC, Woodlands, TX, USA) and HARDENER HV 953 U CI curing agent (Huntsman Petrochemical LLC, Woodlands, TX, USA) were combined in a precise 5:4 ratio by weight at room temperature. The assembly of CFRP and KFRP honeycombs was conducted with the interference fit to achieve better bonding.

### 2.3. Experimental Testing and Characterization

As illustrated in Figure 3d, the crushing experiments were conducted using a WANCE universal electronic testing machine (ETM series, Shenzhen Wance Testing Equipment Co., Ltd., Shenzhen, China). To accurately observe and analyze failure behaviors, all experiments were performed under quasi-static crushing loading conditions, with the rigid plate set at a constant speed of 5 mm/min. The crushing displacement was set to 25–30 mm to ensure that the compaction state was achieved. To minimize experimental errors and enhance accuracy, each sample group was tested three times. Throughout the experiments, the crushing load–displacement relationship was meticulously recorded and their average values were finally calculated. The post-experiment analysis involved using a super-depth-of-field microscope (HR-2016, Hirox Co., Ltd., Tokyo Japan) to observe macro-scale and meso-scale damage and the deformation of samples.

Using a microscope to observe the manufacturing defects on the surface of CFRP and KFRP, it can be found from Figure 3b,c that there is a small amount of dragging out of both carbon and Kevlar fibers in the localized area. Meanwhile, the carbon and Kevlar fibers are exposed due to the local printing defects of Onyx in which the superficial Onyx layer has little effect on the mechanical properties. The contour accuracy of the honeycomb structure is relatively better.

### 2.4. Crashworthiness Indicators

To evaluate the crashworthiness of the carbon/Kevlar hybrid continuous fiber composite honeycomb structure, the main crashworthiness indicators, such as max load and energy absorption (*EA*), are adopted. In addition, the cost of 3D-printed samples is relatively high, and cost-related crashworthiness is also very important, including max load/cost and *EA*/cost. These crashworthiness indicators are introduced as follows.

The max load denotes the peak value in the crushing load–displacement curves. The *EA* denotes the total energy dissipated by the deformation and failure of structures. Both the max load/cost and *EA*/cost denote the corresponding crushing properties per unit cost.

(1) $$EA=\int_{0}^{s}F\left(x\right)dx$$

where *F*(*x*) is the crushing load, *x* is the crushing displacement, and *S* is the total crushing displacement, which denotes the corresponding displacement for the densification stage or global collapse.

### 2.5. Numerical Modeling and Damage Model

**Figure 4:**
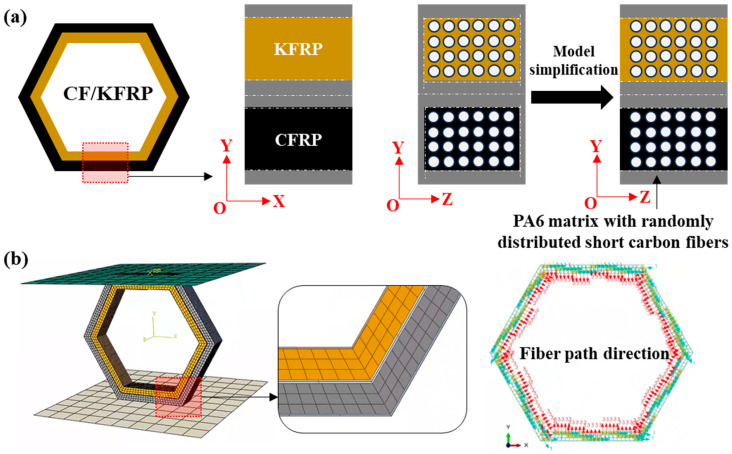
(**a**) Equivalent modeling and model simplification. (**b**) Finite element model with mesh. PA6 denotes Polyamide-6.

The composite honeycomb structure fabricated using a Markforged 3D printer features a small amount of Onyx on both sides in the YOZ coordinate system (CSYS). To facilitate computational analysis, the model can be simplified, as illustrated in Figure 4a. The CF/KFRP composite honeycomb structure comprises CFRP, KFRP, and Onyx. The production of these experimental samples using Markforged 3D printing is both complex and costly. Consequently, finite element analysis (FEA) is employed to predict the crushing performance and analyze the failure mechanisms, substituting for experimental testing.

The finite element model, depicted in Figure 4b, is established to simulate the crushing behavior of the CF/KFRP honeycomb structure. This model includes a deformable honeycomb core and two rigid plates. The dimensions of the model correspond to those of the experimental samples. In the model, the deformable CF/KFRP honeycomb is represented using continuous shell elements (SC8R) with a side length of 1 mm, while the rigid plates are modeled using three-dimensional rigid elements (R3D4) with a side length of 0.5 mm. The fiber direction aligns with the edge length of the honeycomb cells. To accurately simulate the fracture failure of the deformable honeycomb, the element deletion method is implemented. For contact behavior, the Penalty function algorithm with a friction coefficient of 0.1 models the friction interaction among various parts. The “Hard” contact algorithm is utilized to define normal contact behaviors, preventing penetrations. Furthermore, interlaminar delamination is a critical failure mode that influences mechanical properties. Therefore, the Traction–Separation model, in conjunction with the mixed-mode fracture energy method, is employed to capture the cohesive contact behavior between adjacent layers.

This approach leverages the strengths of finite element modeling to provide a detailed understanding of the mechanical performance and failure mechanisms of composite honeycomb structures, complementing and enhancing the findings from experimental methods.

To precisely simulate the damage behavior of CF/KFRP honeycomb structures, the implementation of the maximum stress criterion is essential. This criterion facilitates the accurate prediction of tension and compressive damage in both the matrix and the fibers. To further characterize the material response, the elastic stress–strain relationship for orthotropic damage elasticity, from Matzenmiller et al. [36], is defined using the constitutive relationship delineated in Equation (2).

(2) $$\left[\begin{array}{c} \varepsilon_{11}\\ \varepsilon_{22}\\ \varepsilon_{12}^{el} \end{array}\right]=\left[\begin{array}{ccc} \frac{1}{\left(1-d_{1}\right)E_{1}} & \frac{-v_{12}}{E_{1}} & 0\\ \frac{-v_{12}}{E_{2}} & \frac{1}{\left(1-d_{2}\right)E_{2}} & 0\\ 0 & 0 & \frac{1}{\left(1-d_{12}\right)2G_{12}} \end{array}\right]\left[\begin{array}{c} \sigma_{11}\\ \sigma_{22}\\ \sigma_{33} \end{array}\right]$$

In this model, the damage variables *d*_1_ and *d*_2_ correspond to fiber fractures along the 11- and 22-directions, respectively, while *d*_12_ pertains to matrix microcracks induced by shear deformation. The model distinctly characterizes fiber tension and compressive failure modes by activating the relevant damage variables contingent on the stress state in the fiber direction.

The Hashin failure criterion is adopted to simulate the initial failure of honeycomb, which is shown as follows:

(a) Fiber tension failure;

(3) $$F_{ft}={\left(\frac{\sigma_{11}}{X_{T}}\right)}^{2}+\alpha {\left(\frac{\sigma_{12}}{S}\right)}^{2}\geq 1,$$

(b) Fiber compression failure;

(4) $$F_{fc}={\left(\frac{\sigma_{11}}{X_{c}}\right)}^{2}\geq 1$$

(c) Matrix tension failure;

(5) $$F_{mt}={\left(\frac{\sigma_{22}}{Y_{T}}\right)}^{2}+\alpha •{\left(\frac{\sigma_{12}}{S}\right)}^{2}\geq 1$$

(d) Matrix compression failure;

(6) $$F_{mc}={\left(\frac{\sigma_{22}}{2S}\right)}^{2}+\left[{\left(\frac{Y_{C}}{2S}\right)}^{2}-1\right]•{\left(\frac{\sigma_{22}}{Y_{C}}\right)}^{2}+{\left(\frac{\sigma_{12}}{S}\right)}^{2}\geq 1$$

where α is the shear contribution factor.

The evolution of damage variables is a function of the damage threshold and the fracture energy per unit area. The non-linear evolution equation of damage variables is adopted as follows [37]:

(7) $$d_{\alpha }=1-\frac{1}{r_{\alpha }}\exp\left(-\frac{2g_{0}^{\alpha }L_{c}}{G_{f}^{\alpha }-g_{0}^{\alpha }L_{c}}\left(r_{\alpha }-1\right)\right);\;g_{0}^{\alpha }=\frac{X_{\alpha }^{2}}{2E_{\alpha }},$$

where $L_{c}$ is the characteristic length of the element, $G_{f}^{\alpha }$ is the fracture energy per unit area under uniaxial tension/compressive loading, and $g_{0}^{\alpha }$ is the elastic energy density (i.e., unit volume) at the starting point of failure.

In terms of interlaminar failure, a quadratic nominal stress criterion is adopted to predict the occurrence of delamination as follows [38]:

(8) $${\left(\frac{\langle \sigma_{n}\rangle }{S_{n}}\right)}^{2}+{\left(\frac{\tau_{t}}{S_{t}}\right)}^{2}+{\left(\frac{\tau_{t}}{S_{s}}\right)}^{2}=1,$$

where $\sigma_{n}$, $\tau_{t}$ and $\tau_{s}$ are the contact stress components of the interface layer, respectively, and $S_{n}$, $S_{t}$ and $S_{s}$ are the critical interface strength values, respectively. The linear damage evolution law is used as follows:

(9) $$D=\frac{\delta_{m}^{f}\left(\sigma_{m}^{\max}-\sigma_{m}^{0}\right)}{\sigma_{m}^{\max}\left(\sigma_{m}^{f}-\sigma_{m}^{0}\right)},$$

where $\sigma_{m}^{\max}$, $\sigma_{m}^{f}$ and $\sigma_{m}^{0}$ denote the maximum value of the mixed modal displacement, the effective displacement at full damage, and the effective displacement at the onset of damage, respectively. *d* is a scalar damage variable used to represent the degree of overall delamination damage. D = 0 indicates no damage, 0 < D < 1 indicates partial damage, and D = 1 indicates complete damage.

*G_C_* is the total critical mixed-mode fracture energy, and the expression is presented below [39].

(10) $$G_{n}^{C}+\left(G_{s}^{C}-G_{n}^{C}\right)\left(\frac{G_{S}}{G_{T}}\right)=G_{C},$$

(11) $$G_{S}=G_{s}+G_{t},G_{T}=G_{n}+G_{S}G_{i},$$

where $G_{S}$ and $G_{T}$ refers to the work performed by the traction stresses and their conjugate separations. $G_{n}^{C}$ and $G_{s}^{C}$ are the critical mode-I fracture energy in the normal direction and mode-II fracture energy in the shear direction, respectively, where η is a cohesive property parameter.

## 3. Results and Discussion

### 3.1. Crushing Responses

**Figure 5:**
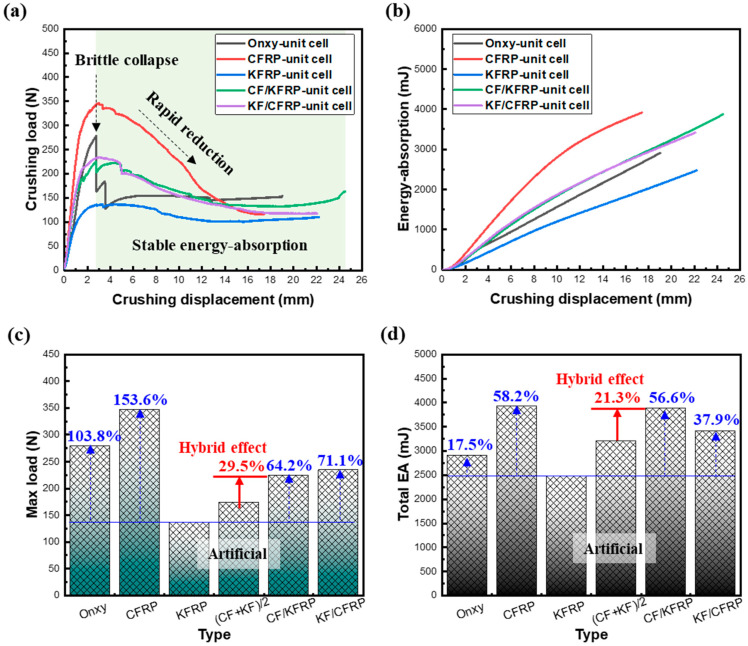
Comparisons of experimental results among four types of honeycombs. (**a**) Crushing load–displacement curves. (**b**) Energy absorption. (**c**) Max load. (**d**) Total EA. (CF + KF)/2 denotes the value of half the sum of CFRP and KFRP, which is used to represent the average value. EA denotes the energy absorption.

Figure 5 shows the comparisons of experimental results among four types of honeycombs. First, it can be observed from Figure 5a that the CFRP honeycomb has the highest crush load and structural stiffness, indicating that it has the highest maximum load-carrying capacity. On the contrary, the KFRP honeycomb has the lowest crush load, with a difference of 153.6% from the highest load of CFRP, which is due to the lower stiffness and strength of Kevlar fibers. Although CFRP honeycomb had the highest load-carrying capacity, the compression collapse load continued to decrease after reaching the peak value, which substantially caused a loss of the load-carrying capacity and reduced the stability. In contrast, the KFRP honeycomb maintained a relatively stable load after reaching the failure load. For the Onyx honeycomb, it is clear that the compression collapse curve suddenly falls off a cliff after the peak, which is due to the fact that Onyx, despite having short carbon fibers inside, still has an insufficient load-carrying capacity compared to continuous fibers. However, the maximum load-carrying capacity of Onyx honeycomb is higher than that of the less-strong KFRP honeycomb.

It can be noticed from the graph that CF/KFRP and KF/CFRP honeycombs have very similar maximum load-carrying capacities, which slowly decrease after reaching the peak value, and then the crushing load becomes smoother as well. In comparison, the load-carrying capacity of CF/KFRP honeycomb with KF/CFRP honeycomb is 64.2% and 71.1% higher than that of KFRP honeycomb. In addition, it can be found that the collapse load curve of KF/CFRP honeycomb ends at a displacement of about 22 mm, which is 2.5 mm earlier than that of CF/KFRP honeycomb. This is due to the difference in the hybrid method, where the CFRP, when located on the inner side, is susceptible to brittle fracture, and then it is difficult to support the KFRP honeycomb on the outer side. On the contrary, when the KFRP honeycomb is located on the inner side, because of its higher toughness, it can still support the failed material of the outer CFRP honeycomb after flexible failure deformation occurs.

### 3.2. Energy Absorption

Energy absorption is an important evaluation index for the mechanical performance of honeycomb structures. As shown in Figure 5b, it can be found that the energy absorbed by the KFRP honeycomb is not significant, being 103.8% lower than that of the Onyx honeycomb. The lack of energy absorption of the Onyx honeycomb is mainly attributed to the brittle fracture after the peak and premature termination of the compression collapse. The CFRP honeycomb, on the other hand, absorbed 58.2% more energy compared to the KFRP honeycomb due to its ability to dissipate a significant amount of energy due to high-strength fracture damage. Both CF/KFRP honeycomb and KF/CFRP honeycomb exhibited higher energy absorption compared to KFRP honeycomb with the blending of carbon fibers with Kevlar fibers, which increased by 56.6% and 37.9%, respectively. This is mainly attributed to the incorporation of carbon fibers into the Kevlar fibers, which improves the strength of the honeycomb structure and absorbs a large amount of energy through the fracture dissipation of the high-strength carbon fibers. However, there is a large influence of the blended form of carbon fibers incorporated into Kevlar fibers. For example, CF/KFRP exhibits a higher energy absorption that is essentially on par with CFRP honeycomb, ensuring that all else is equal, as shown in Figure 5d. This suggests that the addition of a certain amount of high-strength carbon fibers to the Kevlar fibers significantly improves the energy absorption.

### 3.3. Cost Analysis

**Figure 6:**
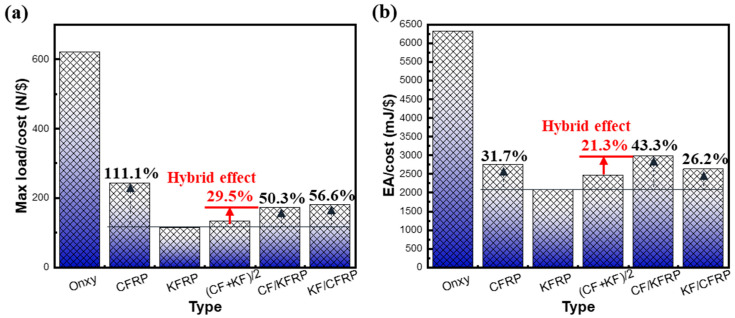
The comparison of the cost of six types of honeycombs. (**a**) Max load/cost. (**b**) EA/cost.

Fiber hybridization is also cost-effective in terms of material cost. According to the material prices issued by Markforged (shown in Table 2), the cCF/O, cKF/O, and Onyx unit prices are USD 0.48, USD 0.31, and USD 0.38$, respectively. The cost of Onyx honeycomb, CFRP honeycomb, KFRP honeycomb, CF/KFRP honeycomb, and KF/CFRP honeycomb can be obtained by slicing tool Eiger calculation, as shown in Table 3. According to this table, it is illustrated that CFRP honeycomb has the highest cost, Onyx honeycomb has the lowest cost, and CF/KFRP honeycomb and KF/CFRP honeycomb are in the middle. Figure 6 visually compares the cost performance of all cellular structures, illustrating that CFRP honeycomb is 31.7% more cost effective than KFRP, while Onyx honeycomb has a high cost performance but not a high overall performance. With the exception of Onyx, CF/KFRP honeycomb and KF/CFRP honeycomb have 43.4% and 26.2% higher cost performances than KFRP honeycomb. The comprehensive analysis shows that CF/KFRP honeycomb not only has a greater advantage in terms of total energy absorption and cost-effectiveness but also that the only shortcoming is that the maximum load-carrying capacity is still weaker than that of CFRP honeycomb. In the subsequent engineering applications, specific composite honeycombs can be rationally designed and selected according to the specific requirements.

In terms of balancing mechanical performance and cost, it can be mainly considered from two dimensions: bearing capacity and cost and energy absorption and cost. From Figure 5 and Figure 6, it can be seen that, if the focus is more on cost factors, short-fiber-reinforced Onyx with moderate performance is a more suitable choice. Although CFRP honeycomb has excellent load-bearing capacity, its cost is high. If the application scenario is critical, the mechanical performance and costs of the main load-bearing components in the aerospace or automotive fields, such as impact or collision-resistant structures, can be balanced by optimizing or adjusting the CF/KFRP fiber ratio to meet the specific application needs. Adding a slight amount of Kevlar fiber to carbon fiber can not only improve the toughness of the material while ensuring its load-bearing capacity but also reduce costs. In the field of military ballistic resistance applications, although KFRP is widely used, its low strength can be moderately enhanced by adding a small amount of carbon fiber, and the cost will only increase slightly. From the perspective of manufacturing costs, although 3D printing technology can be used to manufacture complex composite honeycomb structures, the cost of mass production is relatively high. However, with the development of additive manufacturing technology, the related manufacturing costs will gradually decrease.

### 3.4. Crushing Failure Modes

**Figure 7:**
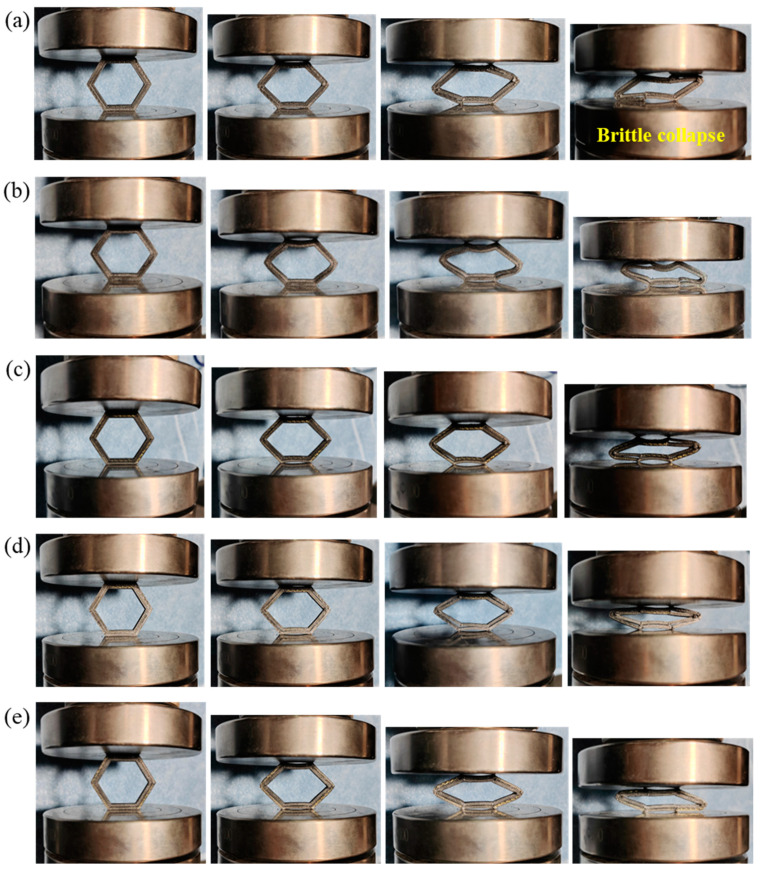
The crushing failure process of the experimental samples. (**a**) Onyx with brittle failure in corners. (**b**) CFRP with brittle failure. (**c**) KFRP with ductile failure. (**d**) CF/KFRP with brittle failure in the CF layer (outer layer) and ductile failure in the KF layer (inner layer). (**e**) KF/CFRP with ductile failure in the KF layer (outer layer) and brittle failure in the CF layer (inner layer).

**Figure 8:**
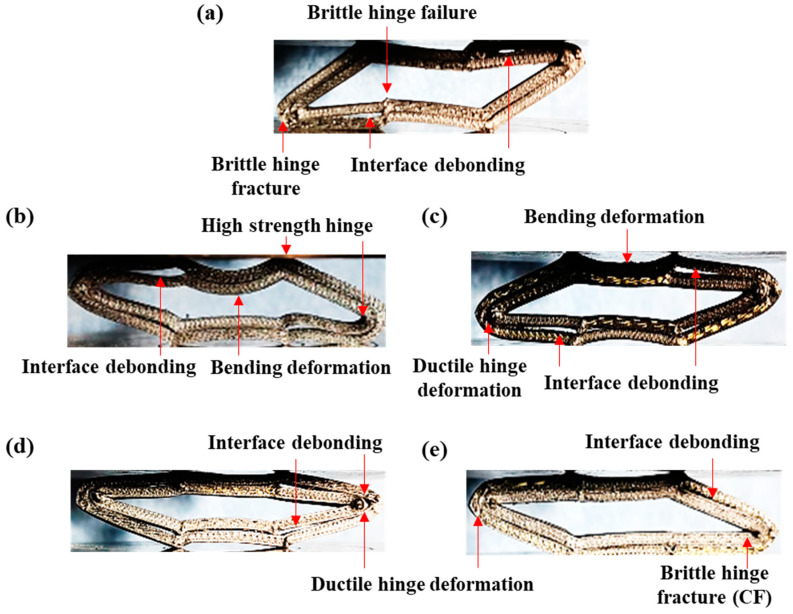
Final crushing failure modes of the experimental samples. (**a**) Onyx with brittle hinge failure and interface debonding. (**b**) CFRP with a high-strength hinge, and local bending deformation. (**c**) KFRP with ductile hinge deformation, bending failure and interface debonding. (**d**) CF/KFRP and (**e**) KF/CFRP with ductile hinge deformation, brittle hinge fracture, and interface debonding.

Figure 7 shows the crushing failure process of the experimental samples. From Figure 7, it can be observed that all composite honeycomb structures were gradually crushed under quasi-static crushing loads and eventually approached the compacted state. Figure 8 demonstrates the final crushing failure modes of the experimental sample. As shown in Figure 8a, the Onyx honeycomb exhibits a brittle fracture collapse, including brittle hinge fracture failure, which is due to the limited reinforcement of the matrix by the short carbon fibers. In contrast, the CFRP honeycomb is strongly reinforced by continuous fibers on top of the short carbon fiber reinforcement, and the whole structure exhibits a behavior with a tougher toughness than the Onyx honeycomb during the compression collapse process. For example, CFRP honeycomb formed a localized bending deformation under the action of high-strength hinges. For the KFRP honeycomb, the Kevlar fibers have higher toughness, and the whole compression collapse process exhibits a flexible collapse accompanied by a flexible hinge deformation, as shown in Figure 8c. For the hybrid fiber composites, the honeycomb exhibits both brittle failure in the CF layer and flexible failure in the KF layer during the collapse process. As shown in Figure 8d, the CF layer in the CF/KFRP honeycomb exhibits brittle failure, but the Kevlar fibers in the inner layer undergo a form of flexible hinge deformation wherein the hinge has not yet been completely broken and can play a certain protective role while continuing to support the CF in the outer layer; on the contrary, as shown in Figure 8e, brittle hinge breakage occurs within the CF layer in the inner layer in the KF/CFRP honeycomb, which reduces the load-transferring efficiency and makes it difficult to support the KF layer in the outer layer. Additionally, the interfacial failure of Onyx/Onyx leads to a reduction in the load transfer efficiency between the CF layer and the KF layer, resulting in a loss of structural performance.

### 3.5. Hybrid Mechanism

The hybrid effect coefficient *H_e_* is a key index used to evaluate the mechanical properties of the hybrid fiber composite honeycomb structure, and it is defined as follows.

(12) $$H_{e}=\frac{P_{h}}{P_{ave}}-1$$

where *P_h_* denotes the performance of the hybrid fiber composite honeycomb. *P_ave_* denotes the average performance values of two types of single-fiber composite honeycomb.

(13) $$\left\{\begin{array}{c} H_{e}>1,\;positive\;hybrid\;effect\\ H_{e}<1,\;negative\;hybrid\;effect \end{array}\right.$$

As shown in Figure 5c,d, it can be found that the CF/KFRP honeycomb and KF/CFRP honeycomb are much higher than the average sum calculated manually, e.g., the maximum load-carrying capacity and the total absorbed energy of the CF/KFRP honeycomb are 29.5% and 21.3% higher. Meanwhile, according to the hybrid effect coefficients given in Table 3, it shows that CF/KFRP honeycomb and KF/CFRP honeycomb exhibit high hybrid effects in terms of maximum load-carrying capacity and energy absorption, especially for CF/KFRP honeycomb.

The reason for the positive hybrid effect in CF/KFRP honeycomb and KF/CFRP honeycomb is mainly attributed to the hybrid mechanism. Discussed in terms of failure modes, the internal failure of the CF layer is brittle fracture, while the internal failure of the KF layer is flexible destructive deformation, and there is an interaction between the two during the compression collapse process. The blending mechanism to improve the honeycomb performance can be explained as follows. The flexible deformation of Kevlar fibers can moderately inhibit the brittle fracture of carbon fibers, thus obtaining a strong–tough balance effect and avoiding the global brittle collapse of the whole honeycomb. Kevlar fibers have good energy-absorbing properties, but the material strength itself is low, which significantly improves the load-bearing capacity of the initial crush or pre-crush phase under the high-strength properties of carbon fibers, which in turn improves the energy absorption. Under the joint action of carbon fibers and Kevlar fibers, the whole honeycomb structure not only has a high load-bearing capacity but also exhibits long-term stable collapse behavior, which ensures that the material can fully dissipate energy through deformation and damage.

### 3.6. Simulation and Failure Analysis

**Figure 9:**
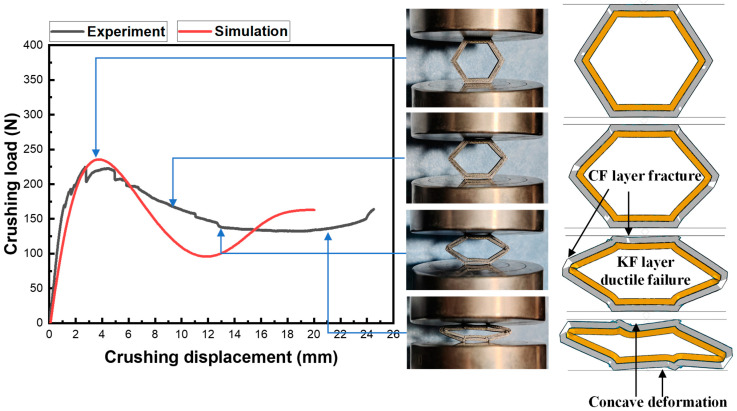
Comparison of crushing load and failure process between experiment and simulation.

The finite element simulation method is used to further carry out the failure analyses of CF/KFRP honeycombs. Figure 9 compares the experimental and simulation results of CF/KFRP honeycombs in terms of crushing load and failure process. From the figure, it can be observed that, at the elastic deformation stage of the curve, the deformation of the simulation and experiment is very small. Then, the peak load is reached, and it can be found that the simulated peak load (235.5 N) agrees well with the experimental peak load (224.6 N), which shows only a difference of 4.9%. At this time, the honeycomb deformation of the experiment and the simulation is relatively obvious, and the outer CF layer fails by local brittle fracturing. After the peak load, the curve gradually decreases and the load-carrying capacity decreases. This is due to some fracture damage in the honeycomb, which briefly lost most of its load-bearing capacity. Subsequently, the load-bearing capacity is recovered again, and, eventually, a stable compression–crush energy absorption is formed. By the experimental and simulated comparison, it is indicated that the average load shows only a difference of 5.8%. For the KF layer, it can be observed that the ductile failure occurs during the whole crushing failure process due to the high toughness of Kevlar fiber. By comparing the experimental and simulated crushing loads and failure processes, it is shown that the simulation and experimental results perform similarly and that further failure analyses could be of some reference.

**Figure 10:**
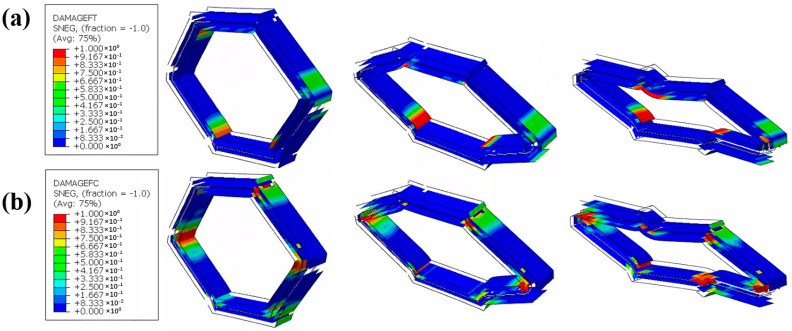
The simulated damage evolution process of CF/KFRP honeycomb. (**a**) Fiber tension damage (DAMAGEFT). (**b**) Fiber compression damage (DAMAGEFC).

**Figure 11:**
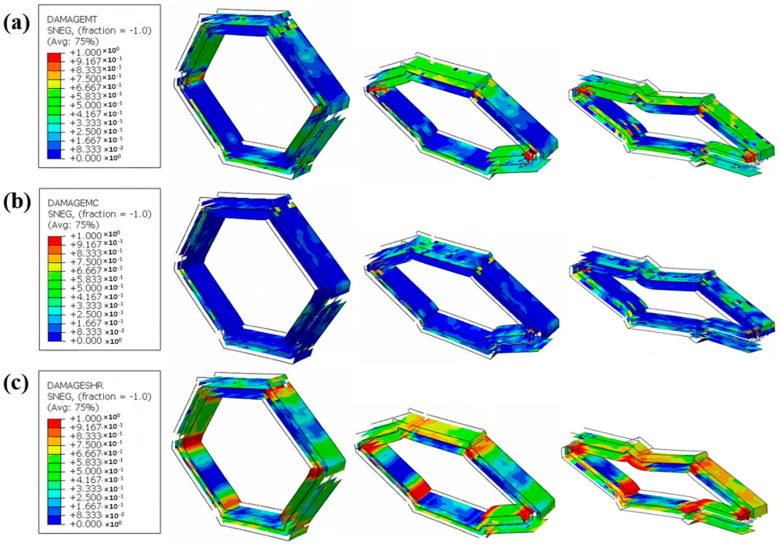
The simulated damage evolution process of CF/KFRP honeycomb. (**a**) Matrix tension damage (DAMAGEMT). (**b**) Matrix compression damage (DAMAGEMC). (**c**) Shear damage (DAMAGESHR).

Figure 10 demonstrates the simulated CF/KFRP honeycomb damage evolution process, including fiber tension damage and compression damage. As shown in Figure 10a, the Kevlar fiber tension damage occurs first in the inner layer of the honeycomb; the Kevlar fiber tension damage then extends rapidly, and the most serious area of fiber tension damage is located at the corner. With the increase in the compression collapse load, carbon fiber tension damage also occurs rapidly in the outer CF layer and brittle fracture occurs at the corners, while the Kevlar fibers maintain a relatively high ductile deformation. For the compression damage of the fibers, similar to the fiber tension damage, the damage area is larger, as shown in Figure 10b. The compression damage of the fibers first started from the lower part of the honeycomb and then rapidly extended to most of the area. Figure 11a–c show the tension, compression, and shear damage process of the matrix. From the overall view of the figures, the matrix tension damage area is larger, significantly larger than the matrix compression damage. The matrix tension damage expands faster, mainly appearing in the outer CF layer. In contrast, the shear damage of the CF layer and KF layer of composite honeycomb are more obvious and appear close to complete damage at the corners (e.g., the dark red area), which also indicates that the shear damage has a greater impact on the structural failure of composite honeycomb.

**Figure 12:**
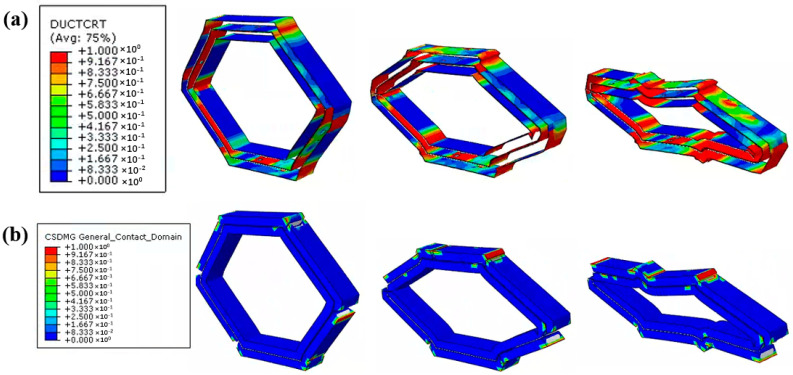
The simulated damage evolution process of CF/KFRP honeycomb. (**a**) Onyx damage (DUCTCRT). (**b**) Interface damage (CSDMG).

Figure 12 demonstrates the damage evolution of Onyx and the interface in CF/KFRP honeycomb, and it can be found that the Onyx damage area in the three layers is very large and that the propagation speed is relatively fast. The large red area in the figure indicates complete damage, which indicates that the strength of Onyx is low and is prone to serious damage during the compression collapse process. Interfacial damage is also very critical for honeycomb bearing. As shown in Figure 12b, Onyx/Onyx interface damage, Onyx/CF interface damage, and Onyx/KF interface damage are included here. From the figure, it is observed that all types of interfacial damages start to sprout from the six corners of the honeycomb and then expand to the periphery. The most serious interfacial damage is located between the CF layer and the KF layer, which is due to the large difference in the stiffness of the two materials and is prone to interfacial debonding.

## 4. Future Works

With the development of 3D printing technology for continuous fiber composites, the mechanical properties of various complex composite structures have drawn widespread attention. Regarding the crashworthiness of 3D-printed hybrid continuous fiber composite honeycomb structures, there are still numerous tasks worthy of in-depth exploration in the future. Firstly, the 3D printing process parameters can be further optimized to enhance the accuracy and quality as well as the overall performance of honeycombs. By precisely controlling parameters, defects in the structure can be reduced and its mechanical properties can be strengthened. Secondly, the impacts of the hybrid ratio and hybrid method of different fibers on the performance of honeycomb structures can be investigated. This can help find the optimal hybrid scheme to achieve higher strength, stiffness, and toughness. Thirdly, in hybrid fiber composites, interface issues are particularly critical. Due to the significant stiffness mismatch between adjacent layers caused by different fibers, interface delamination is prone to occur in the initial stage of failure, which has a significant adverse effect on the overall mechanical properties. Therefore, it is necessary to adopt interface-toughening technology to solve the interface failure problem of hybrid fiber composites. At present, research is mainly centered on composite honeycomb cells. This can be further expanded to improve the crashworthiness of multi-cellular honeycombs and further broaden their application fields in practical engineering. In terms of mechanical modeling, it is possible to establish a composite damage model that takes 3D printing defects into consideration to accurately simulate and evaluate the mechanical properties and failure behavior of 3D-printed composite honeycombs. This can provide a more reliable basis for their design and optimization. In summary, the research on 3D-printed hybrid continuous fiber composite honeycomb structures is filled with challenges and presents opportunities for the future.

## 5. Conclusions

This study primarily investigates the crashworthiness of Markforged 3D-printed hybrid continuous fiber composite honeycomb, incorporating a hybridization of carbon and Kevlar fibers. Through experimental and simulation analyses, the crashworthiness and failure behaviors of hybrid composite honeycombs are examined. By characterizing the crushing failure modes, the energy-absorbing mechanism is uncovered. Subsequently, the hybrid effect and mechanism are elucidated to illustrate the enhancements in crashworthiness. The key findings are summarized as follows.

CFRP honeycomb, despite having the highest load-carrying capacity, folds rapidly post-peak due to the inherent brittleness of carbon fibers. CF/KFRP and KF/CFRP honeycombs, positioned between CFRP and KFRP in terms of load capacity, exhibit relatively stable behavior throughout the crushing load process. CFRP and CF/KFRP honeycombs boast the highest energy absorption capacities, outperforming KFRP honeycomb by 58.2% and 56.6%, respectively. This underscores the significant enhancement in both load-carrying capacity and energy absorption when combining Kevlar fibers with carbon fibers.

In terms of cost, although Onyx honeycomb seems to offer the best performance per cost, its drawbacks make it less appealing. CFRP honeycomb outperforms KFRP by 111.1% in terms of load-to-cost ratio, while CF/KFRP honeycomb provides superior energy absorption for the cost. Hence, CF/KFRP honeycomb holds great promise for energy absorption applications.

CFRP withstands deformation with strong hinges, and CF/KFRP and KF/CFRP exhibit the mixed failure modes. The placement of KF in the inner layer aids in supporting fractured CFs, enhancing energy absorption. Hence, the hybridization in CF/KFRP and KF/CFRP enhances load-bearing, ensuring stable collapse behavior and efficient energy dissipation. This synergy between high-strength carbon and high-toughness Kevlar fibers maximizes their individual properties and fosters additional benefits through interactions.

The simulated and experimental crush response and failure process are in good agreement. The fiber tension damage and fiber compression damage of CF/KFRP honeycomb are more obvious, which have a greater impact on the load-bearing performance of the honeycomb. The damage of Onyx layer is serious and close to the complete damage state. The interfacial debonding mainly occurs at the corners and rapidly expands to the periphery.

## Figures and Tables

**Table 1:** The properties of each raw material [35].

	cCF	cKF	Onyx
Filament type	Continuous carbon fibers embedded in a PA6 matrix	Continuous Kevlar fibers embedded in a PA6 matrix	PA6 reinforced with short carbon fibers
Density (g/cm^3^)	1.4	1.2	1.2
Flexural Strength (MPa)	540	240	71
Flexural Modulus (GPa)	51	26	3.0

**Table 2:** The price of Markforged materials and each honeycomb.

Type	Volume (cc)	Price ($)	Unit Price ($/cc)
Onyx	800	303.6	0.38
cCF/O	1500	717.6	0.48
cKF/O	1500	469.2	0.31
Onyx honeycomb	2.41	0.57	-
CFRP honeycomb	3.79	1.43	-
KFRP honeycomb	3.87	1.19	-
CF/KFRP honeycomb	3.83	1.30	-
KF/CFRP honeycomb	3.83	1.30	-

cc denotes the cubic centimeter. These materials were purchased on 10 May 2023.

**Table 3:** Hybrid effect of CF/KFRP and KF/CFFRP honeycomb.

Type	Max Load			EA		
	*P_ave_* (N)	*P_h_* (N)	*H_e_*	*P_ave_* (mJ)	*P_h_* (mJ)	*H_e_*
CF/KFRP	173.5	224.6	+0.29	3196.9	3877.7	+0.21
KF/CFRP	173.5	234.1	+0.35	3196.9	3413.4	+0.06

## Data Availability

The original contributions presented in this study are included in the article. Further inquiries can be directed to the corresponding author.
